# Effects of residence and race on burden of travel for care: cross sectional analysis of the 2001 US National Household Travel Survey

**DOI:** 10.1186/1472-6963-7-40

**Published:** 2007-03-09

**Authors:** Janice C Probst, Sarah B Laditka, Jong-Yi Wang, Andrew O Johnson

**Affiliations:** 1 South Carolina Rural Health Research Center, Department of Health Services Policy and Management, Arnold School of Public Health, University of South Carolina, Columbia, SC 29208, USA; 2 Department of Health Services Policy and Management, Arnold School of Public Health, University of South Carolina, Columbia, SC 29208, USA; 3 South Carolina Rural Health Research Center, Department of Health Services Policy and Management, Arnold School of Public Health, University of South Carolina, Columbia, SC 29208, USA; 4 Department of Health Services Policy & Management, School of Public Health, China Medical University, 91 Hsueh-Shih Road, Taichung 40402, Taiwan

## Abstract

**Background:**

Travel burden is a key element in conceptualizing geographic access to health care. Prior research has shown that both rural and minority populations bear disproportionate travel burdens. However, many studies are limited to specific types of patient or specific locales. The purpose of our study was to quantify geographic and race-based differences in distance traveled and time spent in travel for medical/dental care using representative national data.

**Methods:**

Data were drawn from 2001 National Household Travel Survey (NHTS), a nationally representative, cross-sectional household survey conducted by the US Department of Transportation. Participants recorded all travel on a designated day; the overall response rate was 41%. Analyses were restricted to households reporting at least one trip for medical and/or dental care; 3,914 trips made by 2,432 households. Dependent variables in the analysis were road miles traveled, minutes spent traveling, and high travel burden, defined as more than 30 miles or 30 minutes per trip. Independent variables of interest were rural residence and race. Characteristics of the individual, the trip, and the community were controlled in multivariate analyses.

**Results:**

The average trip for care in the US in 2001 entailed 10.2 road miles (16.4 kilometers) and 22.0 minutes of travel. Rural residents traveled further than urban residents in unadjusted analysis (17.5 versus 8.3 miles; 28.2 versus 13.4 km). Rural trips took 31.4% longer than urban trips (27.2 versus 20.7 minutes). Distance traveled did not vary by race. African Americans spent more time in travel than whites (29.1 versus 20.6 minutes); other minorities did not differ. In adjusted analyses, rural residence (odds ratio, OR, 2.67, 95% confidence interval, CI 1.39 5.1.5) was associated with a trip of 30 road miles or more; rural residence (OR, 1.80, CI 1.09 2.99) and African American race/ethnicity (OR 3.04. 95% CI 2.0 4.62) were associated with a trip lasting 30 minutes or longer.

**Conclusion:**

Rural residents and African Americans experience higher travel burdens than urban residents or whites when seeking medical/dental care.

## Background

Travel burden is a key element in conceptualizing geographic access to health care. A better understanding of distances and mode of travel for individuals seeking health care is particularly important for vulnerable populations, such as rural residents and racial and ethnic minorities, who are more likely to experience barriers to transportation. Rural residents face travel barriers stemming from distance and the lack of public transportation systems in rural areas. Rural households are more likely than urban households to own at least one car [1]. Rural households tend to make fewer trips per day, but travel 38% more miles [1]. Poorer people living in rural areas travel 59% more miles per day than their urban counterparts [1]. Rural residents unable to own or operate cars often depend on friends and family for transportation, limiting their trip timing, route, flexibility, and preferred mode of travel. This dependence has been shown to be associated with reduced numbers of physician visits for chronic care [2]. Public transportation is limited in rural areas; even in rural households without cars, only 1% of trips are made by public transportation [1]. Rural residents with more complex medical conditions are more likely to travel further for care than those living in urban areas, as are children and older people living in rural areas [3-7]. Compared with persons living in urban areas, rural residents reported longer travel time to see a physician, particularly specialists [8].

Barriers to transportation in rural areas compound access problems traditionally experienced by minorities [9,10]. In both urban and rural areas, minorities are more likely to use public transportation for all non-work related trips, even after adjusting for socioeconomic characteristics [11]. African-Americans report longer travel distances for non-work related trips than whites; Hispanics report that non-work related trips are longer in duration than those made by other racial and ethnic groups [11]. Utilization of health care tends to decrease as the distance traveled to care increases. Uninsured Americans living closer to safety-net providers, for example, report fewer unmet health needs and are more likely to have a usual source of care than those who live further away [12]. Transportation barriers to care are also associated with reduced compliance to treatment regimens and lower rates of preventive care, as well as greater difficulties in accessing emergency health care [13,14].

Most previous studies of travel for care have been limited to specific geographic regions or specific populations such as Medicare beneficiaries [3,15], use of mammogram services [16], rural residents with a diagnosis of human immunodeficiency virus [17], follow up care after a myocardial infarction among patients insured through the Veteran's Administration [5], failure to keep physician appointments [18,19] and use of pharmacy services [20]. To the authors' knowledge, no previous studies have examined travel for medical care using a nationally representative population, and examining actual distance information. The research reported here sought to address this gap by using a transportation planning resource, the National Household Travel Survey, to provide a detailed description of travel to care patterns by residence and race and ethnicity. The purpose of this study is to provide nationally representative estimates of the distance traveled along roads and time spent in travel for medical or dental care, comparing differences among rural and urban residents and by race and ethnicity.

Transportation is linked to health through the concept of access. It is generally accepted that access to health care is an important determinant of health status. Aided by advances in geographic information science and technology, the conceptualization and measurement of access has evolved to include spatial measurement. One of the earliest attempts to model the concept of access was proposed by Andersen [21] as the "Behavioral Model of Health Services Use." Anderson suggested that access was determined by predisposing, enabling, and need-based factors. This was later expanded to classify access as *potential *or *realized *[22]. Penchansky and Thomas [23] described access in five dimensions: availability, accessibility, accommodation, affordability and acceptability. Kahn [24] noted that access measures could be sorted into a two-way framework: potential or realized, and spatial or aspatial. Subsequently, Guagliardo [25] partitioned Penchansky and Thomas' dimensions of access spatially, with availability and accessibility (in a geographic sense) collectively grouped as *spatial accessibility*, with the remaining factors characterized as *aspatial*. Guagliardo [25] also delineated four categories of spatial accessibility measurements: provider-to-population ratios, distances to the nearest provider, average travel impedance to a provider, and gravity models. Talen and Anselin [26] note that differing methods yield differing results, requiring the researcher to choose the measure of accessibility most suited to the service being measured and the way the population is likely to travel to the service. In the present study, we use reported measurements of distance and time traveled for health or dental care purposes as a measure of geographic/spatial accessibility to health care.

"Travel impedance" [25] includes measures of Euclidean (straight-line) distance, travel distance along a given path (over a road network, for instance), or travel time between points. By virtue of their point-to-point nature, travel impedance measures have an advantage over provider/population ratios, as they are able to account for border-crossing behaviors [27,28] and intra-area/local provider variations [25]. Travel time analyses often assume optimal driving conditions, but weather disturbances[29], rural terrain, and urban traffic congestion can all inflate estimated travel times that are based on observed measurements of distance [30]. Travel impedance measurements are particularly appropriate for rural areas, where provider choices are limited and the nearest provider is usually the one most likely to be utilized.

A recent study by Collia, Sharp, and Giesbrecht [31] tapped a resource not previously used by health services researchers, the 2001 National Household Travel Survey (NHTS) conducted by the US Department of Transportation (USDOT). The NHTS is used extensively to plan roads and public transportation. The NHTS constitutes the only nationally representative dataset that includes travel for medical or dental care. Further, it includes many measures not included in previous studies of travel for care, including time spent in travel, mode of travel, and perceived barriers stemming from traffic or road conditions. Collia and colleagues [31] compared travel patterns among younger and older adults using the NHTS. Among their results, Collia and colleagues reported that 1.3% of working age (age 18–64) adults and 2.9% of older (age 65 and over) adults traveled for "medical/dental care." Our research builds on that of Collia and colleagues. Ours is the first study to provide nationally representative estimates of travel distance and travel time to care for rural and urban residents, and among members of racial and ethnic minority groups.

## Method

### Study design and data source

We conducted a cross-sectional analysis of the 2001 NHTS. The NHTS was developed by the US Department of Transportation, with input from the Bureau of Transportation Statistics, the Federal Highway Administration, and the National Highway Traffic Safety Administration. The 2001 NHTS obtained information from a nationally-representative sample of households. Eligible participants were civilian, non-institutionalized persons who considered themselves primary residents of households sampled. Group housing settings were excluded. Types of data collected include information on the purpose, mode, transit time, trip length, and other related aspects of daily trips taken within a 24-hour period.

The survey was designed as a "list-assisted random digit dialing survey" [32], (p.3-3). To draw the sample, all phone numbers in the US were grouped in "100-banks" (lists of numbers for which only the last two digits differ). Numbers were first sorted by Census division and by metropolitan/non-metropolitan area location. Metropolitan areas were then sorted by size. Non-metropolitan areas were sorted by state and within state, by county. A serpentine ordering, north to south and east to west, was used to proceed through counties and states.

The survey took place in multiple stages: an introductory letter mailed to selected households, a screening/recruitment phone call, a travel diary package mailed to participants, and a final phone call to record results of the travel diary. Households were not restricted to those with private vehicles, as use of public transportation, walking and other modes was also of interest. Small cash incentives ($5 US) were provided to enhance response. The travel diary contained instructions for recording travel of each household member over a specified 24-hour period, their "travel day" [32]. Data collection took place from March 2001 through May 2002 to provide a representative year of travel.

The total NHTS data set includes responses from 69,817 households, comprised of a national sample of 26,038 households, plus two add-on surveys conducted for selected states, of 28,899 and 14,880 households, respectivel [32]. The overall response rate for the NHTS was 41% [32]. The low response rate is believed to result from consumer resistance to unsolicited phone calls, language barriers, the multi-stage interview design (respondents could decline or drop out at several points), and the high level of participant burden [33]. Response rates differ significantly by home value, race/ethnicity, the number of adults and presence of married persons in the household, and the type and size of the dwelling. Low socio-economic status was correlated with high response rates for the screener/household-level interview, and with low response rates for the extended/personal-level interview [34]. Survey responses are weighted to account for under-response among specific populations. However, adjustments may not fully compensate for under-represented groups.

### Population

We subset the 2001 NHTS to households in which one member made at least one trip for "medical/dental services." [[32], p. M-40] No finer distinctions, such as between medical and dental, or within categories of medical care, were made by the data collection instrument. We used the term "medical/dental" throughout the paper to indicate the non-specific nature of the trip. The NHTS data described 3,914 trips made by 2,432 households, which were then weighted to provide national estimates. The unit of analysis was the trip.

### Definition of variables

#### Dependent variables

The dependent variables were road distance traveled for medical/dental care and time spent in travel. Distance was recorded as miles along roads from a starting location (which may be home, work or other) to a final destination for that trip. Thus, the NHTS measures one-way distance to care. (For purposes of this manuscript, distances are converted into kilometers in text, while the original units are retained in tables.) Time is recorded as the minutes spent on that trip. To provide context for readers not familiar with transportation patterns, we also provide descriptive statistics for travel to work among the households reporting travel for medical/dental care. Consistent with recent research, we selected 30 miles (48.3 kilometers) [35,36] and 30 minutes per trip [37] as measures suggesting a "high" travel burden. Each of these measures is examined separately in multivariate analysis.

#### Independent variables of key interest

Our study uses the urban/rural variable developed by Claritas, Inc, included with the NHTS to characterize each household. The Claritas variable categorizes geographic units based on population density and proximity to an urban center. First, a 4-square mile grid network was overlaid on the US and, using population estimates from the underlying census block groups, population counts for each square were calculated. Then, to calculate "contextual density," Claritas added the population counts for each grid cell to those of the eight surrounding grid cells, and then divided that by the total area (36 square miles). These "contextual population density" values were then ranked from 0 to 99 to create density centiles. Grid squares with values 19 or less were defined as "rural" by Claritas and by the present study, and all grids with a value of 20 or more were grouped as "urban." Subdivisions within the urban category were not distinguished.

The use of population density and the proximity to an urban/metro area to define level of rurality is common to many classification schemes, including the Rural-Urban Continuum Codes (RUCCs) [38] and the Urban Influence Codes (UICs) [39]. Both RUCCs and UICs are county-based, while the Claritas measure is not derived from political boundaries. The Claritas classification scheme, which uses much smaller geographic units of analysis (4-square mile grid squares), has finer resolution than do county-based codes.

Race and ethnicity is reported as white, African American, Hispanic, and other. The NHTS obtains information on multiple race/ethnicity groups, such as Asian Americans and American Indian/Alaskan Natives. However, persons in these racial and ethnic groups were not sufficiently represented in the travel-for-care population to support independent analysis, and were included in an "other" race and ethnicity category.

#### Control variables

Factors in addition to residence and race/ethnicity are known to influence travel for care. Control variables, held constant in multivariate analysis, were conceptualized at three levels: characteristics of the traveler, the trip, and the community. Traveler characteristics included age, sex, educational attainment, occupation, income, family size, and the presence of "a medical condition that makes it difficult to travel outside of the home" [32] (p. M-9). The NHTS did not obtain information on specific medical conditions. Trip and travel characteristics included day of the week, the time of day, and the mode of travel. Ecological factors were perceived traffic conditions, region, and job density. The perceived traffic conditions variable is based on three NHTS questions about travel problems. Persons who agreed that the price of gasoline, rough pavement, or highway congestion were "very much" or "severe" problems for them were categorized as having traffic problems; others were coded as not having such problems. (The NHTS inquired about a total of 11 potential problems, but only the three items listed were asked of all respondents. Remaining items were randomly asked of 50% of respondents.) Region was based on four major Census regions. Job density refers to the number of jobs per square mile in the Census tract in which the household is located and was a Claritas-developed variable included with the NHTS data set.

### Statistical approach

Descriptive and multivariate analyses were conducted in SAS-callable SUDAAN. All estimates presented are weighted appropriately to reflect the complex NHTS sampling design and yield nationally representative estimates. The need for weighting to account for under-represented groups has already been described. In addition, specific states could purchase larger sample sizes, allowing them to make sub-analyses of interest. Survey weights correct for the over-representation of such states when developing national projections.

## Results

### Overview: trips for medical/dental care

Americans made an estimated 5.9 billion trips for medical/dental care in 2001, projecting the total number of trips to the national population. Women comprised more than half of the population traveling for care (Table 1). Nationally, 13.6% of trips for care were made by persons with a medical condition that limits their ability to travel outside the home. The proportion of trips by travel-restricted persons was statistically equal across rural (16.2%) and urban areas (12.9%, p = 0.1842). Persons with a medical condition were more likely to report traveling as a passenger (50.5%) than were persons without such a condition (31.2%, p < 0.0001).

**Table 1:** Characteristics of trips for medical or dental care, by traveler characteristics, NHTS 2001*

	Distribution, all trips, %	Miles per trip	SE	P value	Minutes per trip	SE	P value
Total	100.00			0.9602			0.0001
White	71.69	10.06	0.38		20.64	0.46	
Black	11.15	9.99	1.01		29.11	1.72	
Hispanic	4.97	10.65	2.54		22.49	2.58	
Other	12.19	10.68	1.22		23.53	2.18	

Rural				< 0.0001			< 0.0001
Total	100.00	17.48	1.11		27.23	1.31	
White	80.79	17.11	1.20		26.36	1.32	
Black	8.55	19.83	2.68		30.19	3.64	
Hispanic	3.46	12.50	4.22		25.26	3.59	
Other	7.20	21.27	6.46		34.43	8.81	

Urban				----			----
Total	100.00	8.31	0.30		20.72	0.52	
White	69.40	7.99	0.33		18.97	0.46	
Black	11.80	8.20	0.87		28.91	1.98	
Hispanic	5.35	10.35	2.93		22.04	3.01	
Other	13.45	9.25	1.08		22.05	2.27	

Age				0.3316			0.3721
0~25	21.47	9.41	0.53		20.86	1.01	
26~50	38.59	10.37	0.43		21.75	0.68	
51~75	30.23	10.55	0.58		22.58	0.67	
76~100	9.71	9.74	1.47		23.99	1.72	

Sex				0.1784			0.0165
Male	37.02	10.51	0.40		22.97	0.61	
Female	62.98	9.95	0.35		21.48	0.52	

Education				0.5499			0.0268
High School or Lower	37.91	10.39	0.57		23.55	0.92	
College	46.07	10.29	0.47		21.48	0.67	
Graduate School	13.68	9.24	0.91		19.98	0.92	
Not Ascertained	2.33	9.01	1.16		20.25	1.69	

Medical Condition that Limits Driving				0.0603			0.0013
Yes	13.56	12.17	1.17		26.47	1.46	
No	86.44	9.84	0.31		21.33	0.49	

Occupation of Head of Household				0.5189			0.0005
Sales or Service	11.71	10.06	0.74		22.05	1.19	
Clerical or Admin. Support	5.71	8.84	0.70		17.96	0.91	
Manufacturing, Construction, Maintenance, or Farming	7.08	10.71	1.20		20.91	1.52	
Professional, Managerial, Technical	15.77	9.95	0.59		20.58	0.78	
Other	59.73	10.29	0.45		22.93	0.61	

Household Income				< 0.0001			0.0007
< $20,000	19.26	10.60	1.02		24.78	1.25	
>$20,000 and < $44,999	26.89	11.14	0.70		23.42	1.19	
>$45,000 and < $69,999	23.62	10.32	0.63		21.18	0.89	
>$70,000	22.46	9.42	0.60		19.07	0.72	
Not Ascertained	7.76	7.29	0.51		21.54	1.07	

Family Size				0.3305			0.0068
< = 2 Family Members	41.89	10.18	0.54		22.75	0.68	
3 Family Members	17.52	10.23	0.75		21.46	1.09	
4 Family Members	19.86	9.04	0.70		18.91	1.01	
>4 Family Members	20.73	11.12	0.84		24.06	1.40	

* Estimates are weighted to be nationally representative; they are based on 3,914 observations.

Nearly all trips were made in a personal vehicle, either car (59.5%), van (15.4%), sports utility vehicle (10.7%) or pickup truck (8.2%). The traveler was a passenger in about a third of all trips. Most trips for medical/dental care used private vehicles; only 2.7% of trips used public transportation, 2.7% walked and 0.7% fell into an "other" category. African Americans (16.5%) and Hispanics (24.0%) were markedly more likely than whites (3.6%) to report traveling for care by public transport or walking (p = 0.0002). In rural areas, public transportation or walking was used for so few trips that valid estimates were not possible.

More than three quarters of trips for medical/dental care took place on a weekday (Table 2). Over 90% of trips were made during business hours, 8 am to 5 pm, with 6.3% made between midnight and 8 am, and 3.2% made between 5 pm and midnight (Table 2). About a quarter of trips for medical/dental care involved travelers who agreed that the price of gasoline, rough pavement, or highway congestion were "very much" or "severe" problems for them (Table 2). Rural residents were significantly more likely to perceive the price of gasoline as a problem than were urban residents (27.5% versus 21.5%; p = 0.0075); urban residents were more likely to perceive highway congestion as a problem (21.9% versus 11.5%, p < 0.0001).

**Table 2:** Travel for medical or dental care, by trip and community characteristics, NHTS 2001*

	Distribution all trips, %	Miles per trip	SE	P value	Minutes per trip	SE	P value
**Characteristics of Trip**							

Mode of Travel				< 0.0001			0.0069
Personal Vehicle	93.78	10.56	0.33		21.57	0.47	
Public/Walk/Other	6.22	4.08	0.65		28.96	2.68	

Driver/Passenger Status				< 0.0001			< 0.0001
Passenger	33.75	12.03	0.56		23.49	0.76	
Not Passenger	60.12	9.76	0.29		20.52	0.40	
Public/Walk/Other	6.13	3.74	0.62		28.79	2.70	

Day of Week				0.1540			0.5368
Business Day (Mon.- Friday)	76.44	9.86	0.34		21.88	0.54	
Weekend (Saturday- Sunday)	23.56	11.11	0.78		22.53	0.93	

Time of Day				< 0.0001			0.0001
Midnight – 8 am	6.25	16.93	2.20		30.18	2.97	
Business Hours (8 am – 5 pm)	90.53	9.82	0.33		21.65	0.51	
5 pm – Midnight	3.22	6.55	0.89		16.79	1.74	

**Characteristics of Community**							

Traffic Conditions				0.7084			0.2450
Yes	26.42	9.99	0.50		22.71	0.67	
No	73.58	10.22	0.37		21.79	0.56	

Region				< 0.0001			0.0081
Northeast	19.45	7.10	0.49		19.86	0.83	
South	21.78	10.49	0.51		20.59	0.70	
Midwest	35.49	11.56	0.56		23.32	0.69	
West	23.28	10.26	0.88		23.22	1.44	

Job density				< 0.0001			0.0007
Low	22.33	16.42	1.03		26.12	1.24	
Median	24.52	9.73	0.62		21.32	1.26	
High	24.63	9.18	0.68		21.27	1.10	
Very High	28.53	6.47	0.35		20.10	0.63	

* Estimates are weighted to be nationally representative; they are based on 3,914 observations.

### Distance traveled for medical/dental care

Across the US, the average road distance traveled for medical/dental care was 10.2 miles (16.4 km). Rural residents traveled significantly further for care than did urban residents, 17.5 versus 8.3 miles (28.2 versus 13.4 km; Table 1). The disparity of the distance traveled for care for rural residents was greater than that for work, 15.6 versus 11.6 miles (25.1 versus 18.7 km; Table 3). The presence of a medical condition that limited individuals' ability to travel did not statistically affect distance traveled for those with such a condition versus those without restrictions (Table 1). Mean distance traveled for routine medical/dental care did not differ significantly by race (Table 1). For work-related travel, African Americans traveled slightly shorter distances than whites (11.0 versus 12.6 miles; 17.7 versus 20.3 km, p < 0.001), but other minorities did not differ from whites (Table 3).

**Table 3:** Average time and distance for a trip to work, by residence and race, NHTS 2001*

	Distance Traveled to Work			Time Spent in Travel for Work		
**Distance traveled**	Miles	SE	P-value**	Minutes	SE	P-value**

Total						
Race			0.0026			< 0.0001
White	12.57	0.15	----	22.92	0.21	----
African American	11.01	0.37	0.0002	24.80	0.63	0.0025
Hispanic	12.09	1.25	0.7016	23.53	1.01	0.5414
Other	12.86	0.55	0.5983	25.53	0.65	0.0001

Residence			< 0.0001			0.9709
Rural	15.56	0.35	< 0.0001	23.48	0.43	0.9709
Urban	11.62	0.17	----	23.47	0.23	----

* Estimates are weighted to be nationally representative; they are based on 3,914 observations.

**First indicated p value tests overall siginificance of the variable. Subsequent p-values note differences between individual values and the reference value.

### Time spent in travel for care

The average trip for medical/dental care took 22.0 minutes, comparable to the amount of time persons in the same households spent in traveling to work, 23.5 minutes. Rural trips for medical/dental care averaged 31.4% longer than urban trips, 27.2 versus 20.7 minutes (Table 1). The time spent in work travel was the same for both urban and rural households, 23.5 minutes (Table 3).

Travel time for medical/dental care differed significantly by race, with African Americans reporting significantly longer travel times than whites (29.1 minutes versus 20.6 minutes, Table 1). Other minorities did not differ from whites. Individuals with a medical condition that limited their ability to travel averaged 26.5 minutes for travel, versus 21.3 minutes for travelers without a medical condition (p = 0.0013, Table 1).

As reported in Table 2, how and when a trip for care was made influenced travel time. People who used public transportation or walked to care spent the greatest time in travel (28.8 minutes), followed by those who traveled as a passenger in a personal vehicle (23.5 minutes) and individuals who drove themselves to care (20.5 minutes; p < 0.0001). People spent the greatest time traveling for care if their trip began between midnight and 8 am (30.2 minutes). A trip that began during normal business hours averaged 21.7 minutes, a trip that initiated between 5 pm and midnight averaged 16.8 minutes (p < .0001).

### High Travel Burden – More than 30 Miles (48.3 Kilometers) or 30 Minutes Per Trip

#### Trips for medical/dental care of 30 Miles (48.3 km) or more

Overall, 7.9% of persons seeking medical/dental care traveled 30 road miles (48.3 km) or more. Rural residents were more likely to travel 30 miles or longer for care than urban residents (21.4% versus 4.5%, p < 0.0001, Table 4). Even after controlling for characteristics of the trip and of the community, rural residents were still more likely to experience lengthy trips (Odds Ratio, 2.67, 95% confidence interval, 1.39–5.15; Table 5). Race/ethnicity was not a significant predictor of a trip of 30 miles or more, with other characteristics held constant. No other personal characteristics of the traveler affected the risk for traveling more than 30 miles. Trips taken in the evening (5 pm through 12 midnight) had reduced odds for long travel compared to trips during business hours; trips at night (midnight to 8 am) were more likely to entail long travel (Table 4).

**Table 4:** Proportion of persons with a high travel burden for medical/dental care, by residence and race, NHTS 2001. * (Note: The value for rural, Hispanic travelers is statistically unreliable due to small sample size.)

Percent of trips that are 30 or more miles (46.3 km)							
	Total		Rural		Urban		

	Percent	SE	Percent	SE	Percent	SE	P-values for residence

Total	7.91	0.72	21.44	2.45	4.50	0.70	< 0.0001
White	7.46	0.82	21.53	2.81	3.33	0.58	< 0.0001
African American	6.71	1.91	23.35	9.47	3.67	1.44	0.0484
Hispanic	11.68	5.15	*6.40*	*6.02*	12.54	5.95	0.4777
Other	10.08	3.54	25.42	12.85	8.01	3.79	0.2201
P values for race	0.6461		0.3560		0.2434		

Percent of trips that are 30 minutes or more							

	Total		Rural		Urban		

	Percent	SE	Percent	SE	Percent	SE	P-values for residence

Total	28.54	1.33	41.26	2.99	25.33	1.49	< 0.0001
White	24.41	1.32	38.45	2.98	20.29	1.43	< 0.0001
African American	49.71	4.85	54.87	16.35	48.77	5.09	0.6933
Hispanic	32.86	7.48	*56.09*	*19.37*	29.06	8.60	0.2241
Other	31.68	4.09	49.43	10.14	29.28	4.60	0.1100

P values for race	< 0.0001		0.4320		0.0001		

*Estimates are weighted to be nationally representative; they are based on 3,914 observations.

**Table 5:** Factors associated with a high burden of travel for medical/dental care, separate logistic regression analyses, NHTS 2001.

	Travel of 30 miles or more ^a^		Travel of 30 minutes or more ^b^	
	OR	95% CI	OR	95% CI

**Characteristics of Traveler**				

Residence				
Rural	2.67**	1.39, 5.15	1.80*	1.09, 2.99
Urban (ref)	---	---	---	---

Race				
White (ref)	---	---	---	---
Black	1.03	0.53, 2.02	3.04***	2.00, 4.62
Hispanic	2.45	0.51, 11.77	1.23	0.56, 2.69
Other	1.90	0.69, 5.19	1.64*	1.07, 2.51

Age				
0~25	1.32	0.73, 2.36	0.95	0.70, 1.31
26~50 (ref)	---	---	---	---
51~75	1.31	0.73, 2.35	1.05	0.79, 1.40
76~100	1.59	0.68, 3.69	1.18	0.77, 1.83

Sex				
Male (ref)	---	---	---	---
Female	1.08	0.80, 1.46	0.83	0.68, 1.00

Education				
High School or Lower	1.10	0.59, 2.04	0.94	0.71, 1.24
College (ref)	---	---	---	---
Graduate School	0.63	0.25, 1.61	0.95	0.60, 1.51
Not Ascertained	0.65	0.15, 2.78	0.63	0.32, 1.27

Medical Condition that Limits Driving				
Yes (ref)	---	---	---	---
No	0.64	0.39, 1.04	0.82	0.60, 1.11

Occupation of Head of Household				
Sales or Service (ref)	---	---	---	---
Clerical or Administrative Support	0.52	0.19, 1.39	1.11	0.71, 1.71
Manufacturing, Construction, Maintenance, or Farming	1.23	0.47, 3.26	0.84	0.49, 1.45
Professional, Managerial or Technical	1.02	0.48, 2.17	1.25	0.85, 1.86
Other	0.84	0.46, 1.55	1.20	0.85, 1.70

Household Income				
< $20,000 (ref)	---	---	---	---
>$20,000 and < $44,999	1.18	0.54, 2.61	0.97	0.65, 1.45
>$45,000 and < $69,999	0.90	0.43, 1.87	0.78	0.52, 1.16
>$70,000	1.17	0.51, 2.69	0.68	0.42, 1.09
Not Ascertained	0.30	0.09, 0.93	0.89	0.53, 1.51

Family Size				
< = 2 Family Members (ref)	---	---	---	---
3 Family Members	0.98	0.46, 2.10	0.96	0.64, 1.45
4 Family Members	0.80	0.39, 1.63	1.02	0.67, 1.54
>4 Family Members	1.29	0.62, 2.68	1.44	0.96, 2.18

**Characteristics of Trip**				

Mode of Travel				
Personal Vehicle (ref)	---	---	---	---
Public/Walk/Other	0.40	0.13, 1.26	2.22***	1.42, 3.46

Day of Week				
Business Day (Monday- Friday) (ref)	---	---	---	---
Weekend (Saturday-Sunday)	1.43	0.88, 2.34	1.16	0.85, 1.59

Time of Day				
Midnight – 8 am	2.54*	1.12, 5.78	1.86*	1.12, 3.10
Business Hours (8 am – 5 pm) (ref)	---	---	---	---
5 pm – midnight	0.20***	0.09, 0.43	0.71	0.31, 1.61

**Characteristics of Community**				

Traffic Condition				
Yes (ref)	---	---	---	---
No	0.83	0.58, 1.20	0.81	0.64, 1.02

Region				
Northeast (ref)	---	---	---	---
South	0.98	0.47, 2.06	1.24	0.87, 1.78
Midwest	1.46	0.76, 2.80	1.38	0.97, 1.95
West	2.11	0.88, 5.05	1.40	0.98, 2.00

Job Density				
Low (ref)	---	---	---	---
Median	0.52	0.23, 1.16	0.94	0.58, 1.53
High	0.49	0.20, 1.19	0.86	0.50, 1.46
Very High	0.19**	0.07, 0.52	0.62	0.37, 1.03

^a ^Model Statistics for distance traveled: R2 = 0.0899.

^b ^Model Statistics for time spent in travel: R2 = 0.0817

* p > 0.05. ** p > 0.01. *** p > 0.001.

#### Trips for medical/dental care of 30 minutes or more

Nationally, 28.5% of trips for medical/dental care took 30 minutes or more (Table 4). About two of every five rural residents (41.3%), versus 25.3% of urban, spent more than 30 minutes in travel (p < 0.0001; Table 4). Among urban residents, African Americans and other minorities were markedly more likely to report a trip for medical/dental care lasting 30 minutes or more (Table 4).

As shown in Table 5, rural residents were more likely than urban residents to travel more than 30 minutes for care (OR, 1.80, CI 1.09–2.99), even after controlling for other factors. African Americans (3.04, 95% CI 2.00–4.62) and persons of "other" race (OR 1.64, 95% CI 1.07–2.51) were markedly more likely than whites to have trips for care that required more than 30 minutes of travel.

People relying on public transportation, walking or other modes were more likely than persons traveling in a personal vehicle to spend more than 30 minutes traveling for care (OR 2.22, CI 1.42–3.46; Table 5). Trips made at night (midnight to 8 am) were more likely to take more than 30 minutes than trips made during business hours (OR 1.86, CI 1.12, 3.10). No community characteristics were significantly linked to travel time in multivariate analysis.

## Discussion

The present study is the first to examine and quantify rural-urban differences in travel time and road miles traveled for medical/dental care using a nationally representative sample of households, the US National Household Transportation Survey. With this source, we were able to use actual travel distance, rather than mid-point to mid-point of geographic grids, to define distance. Using this resource, we were able to document the effects of rural residence and race/ethnicity on travel burden.

Rural residents, on average, traveled about eight miles further for care than urban residents, taking about six additional minutes to complete their trip. The proportion of persons traveling 30 miles (48.3 kilometers) or more for medical/dental care was four times higher among rural than among urban residents. Time differences were less pronounced. Rural residence remained an independent risk factor for a high travel burden measured by miles or time, even after controlling for other characteristics of the traveler. These results are consistent with previous studies, using specific populations [3,5,19]. Longer travel distances and times appear to be an inherent element of rural life, as we found similar patterns for travel to the workplace.

Our results highlighted travel disadvantages experienced by African Americans. Half of the trips made by African Americans for medical/dental care took 30 minutes or more, versus 25% for whites. While African Americans also spent more time in travel for work than did whites, the magnitude of such differences was smaller (Tables 1 and 3). The higher proportion of African Americans using public transportation may contribute to lengthy travel times. However, even controlling for mode of transportation, personal characteristics, and community factors, African Americans were more likely to experience a high travel time burden for medical/dental care. Our findings for African Americans are consistent with earlier research [11]. Use of public transportation was independently associated with increased odds for a trip of over 30 minutes in multivariate analysis. These findings suggest that African Americans seeking health care and using public transportation would be particularly disadvantaged in the time expenditure required.

We cannot ascertain, from the data available, whether differences between African Americans and whites in the time burden of travel for medical/dental care stem from patient choice, provider availability, or other locational characteristics not measured in the NHTS. Minorities may elect relatively long trips to visit desired providers. Alternatively, African Americans may have a more limited provider base to choose from, requiring more time-consuming trips. The latter hypothesis seems appropriate for further study, given research noting that the African American population is more likely to be uninsured or to have public insurance [40], [12]. The number of providers willing to accept such patients is substantially lower than the number of those willing to accept private insurance payments [41-43]. Additional population characteristics with spatial dimensions may also influence travel time for African Americans. As noted, African Americans were more likely than whites to report using public transportation. While mode of transport was held constant in our analysis, factors such racial or ethnic residential segregation, leading to differential public transport availability and scheduling, may influence travel times among this population group. The NHTS public use data set does not provide detailed location information; thus, we could not address these important considerations. Future work, using a broader range of ecological characteristics than was available in the present study, may be able to clarify factors associated with African American travel disadvantages.

Several limitations should be weighed when evaluating the results of our research. First, we used a transportation planning instrument as our data source and are restricted to its survey questions. Grouping all travel for care as "medical/dental," while presumably adequate from the point of view of transportation planning, is overly simplistic for health services research. Medical travel is influenced by type of care sought; individuals will travel further for specialty than for primary care [7,17,44,45] and for complex medical cases than for simpler health problems [7]. These distinctions are not captured in the NHTS data set. Second, the NHTS only captures information for persons who *completed *a trip. Thus, it excludes people who defer or avoid seeking medical/dental care, whether because of anticipated travel burden or for other reasons. Third, the NHTS included relatively few Hispanic households with travel for medical/dental care. While this may reflect better health and/or reduced use of care, it may also stem from under-representation of Hispanics in the data. Fourth, the NHTS public use files employ a Claritas proprietary variable to define "rural." This unique definition of rural limits comparability with previous research. Next, we also acknowledge that NHTS response rate is relatively low (41%). Although we included survey weights to address this data characteristic, we acknowledge that the adjustment may not fully account for differential response rates across populations. Notwithstanding the limitations listed above, the NHTS constitutes the only known nationally representative, population based, data source for road-based travel distance and time for medical/dental care.

One cannot study travel for care without commenting on the recent increases in the cost of gasoline in the United States. In 2001, when the NHTS was administered, retail gasoline prices averaged $1.46 per gallon. At that price, about a quarter of persons making trips for medical/dental care reported that the price of gas was a problem for them. As of August 28, 2006, the national average retail price of gasoline had nearly doubled to $2.85 [46]. While prices have declined since that time, continuing fluctuations in response to natural disasters and other constraints on supply may be anticipated. Rural populations have few alternatives to personal vehicles; public transport is seldom available and walking or biking is prohibited by distance and road design. Thus, to the extent that rising gas prices will constitute a barrier to travel for care, rural and poorer populations will be the first to defer or avoid care.

## Conclusion

Addressing differences in travel burden for care based on residence and race/ethnicity will require health planners to work closely with transportation agencies. In urban areas, access to public transportation (routes, hours, frequency of transport) clearly needs to be assessed when planning safety net facility hours and locations. For rural areas, the implications are less clear. Rural travel is overwhelmingly private vehicle travel, and public transportation infrastructure is sparse. Coordination with local transportation planners might reveal location patterns that could be explored, perhaps locating health care services on routes heavily used for work or shopping, allowing rural residents to meet multiple purposes when traveling. Policies should explore additional transportation strategies in rural areas.

## Competing interests

The author(s) declare that they have no competing interests.

## Authors' contributions

JCP conceived of the study, supervised the analysis, and developed the initial draft of the present manuscript. SBL assisted in study design and supervision, and critically revised the manuscript. J-YW participated in study design, carried out the statistical analyses, and contributed to the final manuscript. AOJ participated in study design, developed the literature review, and contributed to the final manuscript. All authors jointly developed conclusions. All authors contributed to the revisions of the manuscript.

## Pre-publication history

The pre-publication history for this paper can be accessed here:


